# Therapeutic Strategies and Genetic Implications for Periodontal Disease Management: A Systematic Review

**DOI:** 10.3390/ijms25137217

**Published:** 2024-06-29

**Authors:** Alessandra Laforgia, Alessio Danilo Inchingolo, Fabio Piras, Valeria Colonna, Roberto Vito Giorgio, Claudio Carone, Biagio Rapone, Giuseppina Malcangi, Angelo Michele Inchingolo, Francesco Inchingolo, Andrea Palermo, Gianna Dipalma

**Affiliations:** 1 Department of Interdisciplinary Medicine, University of Bari “Aldo Moro”, 70124 Bari, Italy; alessandra.laforgia@uniba.it (A.L.); or a.inchingolo1@studenti.uniba.it (A.D.I.); or valeria.colonna@uniba.it (V.C.); or robertovito.giorgio@uniba.it (R.V.G.); or claudio.carone@uniba.it (C.C.); or biagio.rapone@uniba.it (B.R.); or giuseppina.malcangi@uniba.it (G.M.); or a.inchingolo3@studenti.uniba.it (A.M.I.); or gianna.dipalma@uniba.it (G.D.); 2 College of Medicine and Dentistry, CoMD Birmingham Campus, Birmingham B4 6BN, UK; andrea.palermo2004@libero.it

**Keywords:** microbiota’s composition, microorganisms, periodontal disease, surgery, treatment

## Abstract

The objective of this review is to identify the microbiological alterations caused by various therapy modalities by critically analyzing the current findings. We limited our search to English-language papers published between 1 January 2004 and 7 May 2024 in PubMed, Scopus, and Web of Science that were relevant to our topic. In the search approach, the Boolean keywords “microbio*” AND “periodontitis” were used. A total of 5152 papers were obtained from the databases Web of Science (2205), PubMed (1793), and Scopus (1154). This resulted in 3266 articles after eliminating duplicates (1886), and 1411 entries were eliminated after their titles and abstracts were examined. The qualitative analysis of the 22 final articles is included in this study. Research on periodontal disease shows that periodontitis alters the oral microbiome and increases antibiotic resistance. Treatments like scaling and root planing (SRP), especially when combined with minocycline, improve clinical outcomes by reducing harmful bacteria. Comprehensive mechanical debridement with antibiotics, probiotics, EMD with bone grafts, and other adjunctive therapies enhances periodontal health. Personalized treatment strategies and advanced microbial analyses are crucial for effective periodontal management and antibiotic resistance control.

## 1. Introduction

### 1.1. Periodontal Disease

The chronic inflammatory illness called periodontitis damages the structures that support the teeth, progressively destroying the alveolar bone and the periodontal ligament and causing periodontal pockets to form [1]. Many genetic, environmental, and behavioral factors are among the many complex and multidimensional elements influencing this illness [2]. Frequently occurring risk factors encompass genetic predisposition, stress, diabetes mellitus, smoking, and poor oral hygiene [3].

The intricate relationship between the host’s immune system and the pathogenic bacteria found in dental plaque is the fundamental cause of periodontitis [4]. Dental plaque is a biofilm mainly made up of bacteria that builds up on the surfaces of teeth [5]. If it is not removed properly, it can calcify and create calculus [6].

The development of the condition is closely linked to the existence of specific periodontal infections, including *Tannerella forsythia*, *Treponema denticola*, and *Porphyromonas gingivalis*. These bacteria can cause inflammation, weaken the host’s immune system, and harm periodontal tissues because they release a variety of virulence factors [7,8].

The significance of the oral microbiota in the development and course of periodontitis has been demonstrated by recent studies on the oral microbiome [9]. A wide variety of microorganisms, including bacteria, fungi, viruses, and archaea, make up the oral microbiota [10]. This is a very dynamic microbial community that differs greatly between people who have periodontitis and those who do not, as well as across various parts of the same mouth [11,12,13,14,15,16,17,18].

A healthy oral microbiota is typified by a balance (homeostasis) between pathogenic and commensal microorganisms [19]. Commensal bacteria, including those in the *Actinomyces* and *Streptococcus* genera, are crucial for preserving oral health because they inhibit the growth of harmful bacteria and regulate the immune system [20]. Microbial dysbiosis results from this balance being upset, which happens in periodontitis [21]. Pathogenic bacteria proliferate during dysbiosis, which exacerbates inflammation and tissue damage [22,23,24,25,26,27].

Studies using metagenomic and metatranscriptomic approaches have demonstrated that periodontitis is linked to modifications in the microbiome’s functionality as well as an increase in the number of pathogenic bacteria [28].

In order to cause inflammation and trigger the generation of metabolites, toxins, and degradative enzymes that harm periodontal tissues, periodontal pathogens have the ability to change the gene expression of microbial communities [29,30].

Furthermore, the oral microbiota is fundamentally modulated by the host immunological response. The inflammatory response, which is mediated by pro-inflammatory cytokines, including TNF-α, IL-6, and IL-1β, can worsen dysbiosis by encouraging the growth of harmful microorganisms [31]. As a result, inflammation and dysbiosis feed off one another, creating a vicious cycle that eventually makes periodontitis worse [32].

Novel avenues for the diagnosis and management of periodontitis have been made possible by the examination of the oral flora [33].

Modern methods like bioinformatic analysis and high-throughput DNA sequencing enable the identification of certain microbial profiles linked to various illness phases [34,35]. This can help in the creation of individualized therapies meant to improve therapeutic results and restore microbial balance [36].

Therefore, creating more potent treatment plans requires an understanding of the role that the oral microbiota plays in the genesis and progression of periodontitis [37]. After both non-surgical and surgical therapies, the oral microbiota might be disturbed, which can offer new insights into managing the illness and preventing recurrences [38].

### 1.2. Treatment of Periodontitis

There are two types of treatments for periodontitis: non-surgical and surgical. Each has a set of indications and methods of operation [39]. The non-surgical technique, which is regarded as the initial therapeutic strategy, mainly focuses on the mechanical removal of calculus and supra- and subgingival plaque using scaling and root planing (SRP), often referred to as root planing and debridement, in conjunction with oral hygiene education (OHI) [40,41]. The goal of these treatments is to get rid of the germs that cause periodontal inflammation [42]. To further lower the bacterial burden and improve the results of mechanical cleaning, topical or systemic antimicrobial medicines are frequently used in addition to non-surgical treatment [43,44,45].

Live bacteria called probiotics support the health of the host, particularly the mouth cavity, when they are in balance [46]. Probiotics work with oral bacteria to share resources and space, which stops them from colonizing and growing when treating periodontitis. To stop bacteria from growing, they create antimicrobial compounds, such as hydrogen peroxide, organic acids, and bacteriocin [47]. Probiotics have the ability to influence immune response modulation, which, in turn, stimulates the anti-inflammatory response and lessens tissue damage and gingival inflammation [48,49].

Non-viable probiotics, or paraprobiotics, are dormant bacteria, yet they have the capacity to communicate with the immune system and have advantageous effects akin to those of live probiotics [50]. Immunocompromised patients benefit most from the usage of paraprobiotics. By modifying the immune response, paraprobiotics can lessen inflammation and enhance tissue regeneration [51]. Furthermore, they have the ability to stop infections from sticking to oral tissues, lowering the chance of recurring infections.

Bioactive metabolites known as postbiotics are created by probiotics as they develop and undergo metabolic processes [52]. They are essential for regulating the bacteria in the mouth and maintaining dental health. They have the ability to lessen gingival irritation and stop bacterial growth. Tissue filtration can be enhanced and bacterial plaque can be broken down by postbiotic enzymes and peptides [53]. Additionally, by fortifying the oral epithelium, they can enhance local immunity and stop bacterial invasion.

Ozonized materials, including ozonized oil and water, are used to treat periodontitis because of their potent antibacterial and anti-inflammatory properties [54]. Harmful bacteria, viruses, and fungi can be swiftly eliminated by strong oxidants like ozone (O_3_) without causing significant damage to human tissue. During periodontal therapy, ozonized water can be used to irrigate periodontal pockets, which reduces the bacterial burden and promotes tissue healing [55]. Ozonated oil can be applied topically to inflamed gums to reduce swelling and speed up the healing process. Clinical research has shown that ozonized materials significantly improve periodontal parameters such as gingival bleeding and pocket depth [56]. When these therapies are combined, patients with periodontitis may have significant improvements in their oral health and overall quality of life [57].

An inventive and multifaceted strategy for treating periodontitis is provided by the incorporation of probiotics, paraprobiotics, postbiotics, and ozonated substances into non-surgical periodontal therapy. These substances influence the host’s immune system, encourage tissue regeneration, and lower the number of harmful germs. Patients with periodontitis may see notable improvements in their quality of life and periodontal health if these treatments are implemented.

Non-surgical treatment might not always be enough, particularly when there are large bone abnormalities or extensive periodontal pockets present [58]. Surgical therapies are used in these cases, and they involve a number of operations meant to reduce pocket depth, regenerate lost periodontal tissues, and reshape bone abnormalities [59]. Access flaps, restorative surgery, and regenerative treatments such as bone grafting, barrier membranes (guided tissue regeneration, or GTR), and growth factor administration are examples of common surgical approaches [60]. By fostering an oral environment that supports tissue regeneration and upholds a balanced microbial flora, these therapies are intended to lower the chance of illness recurrence [61].

Recent research has shown that the oral microbiota can undergo substantial alterations as a result of both surgical and non-surgical therapies [62]. However, based on the specific treatment plan and the unique characteristics of each patient, the kind and degree of these changes can differ significantly [63,64,65,66,67]. For example, non-surgical therapy usually lowers the total number of bacteria in the mouth and changes the makeup of the oral microbiota, promoting the growth of good bacteria at the expense of periodontal pathogens [68]. However, surgical intervention can also more significantly alter the structure of the microbiota, leading to more favorable and long-lasting recolonization, in addition to decreasing pocket depth [69].

Comprehending the impact of these therapies on the oral microbiota is essential for refining therapeutic approaches and enhancing enduring clinical results [70]. In order to maintain periodontal health and stop recurrences, a healthy oral microbiome is necessary [71]. As a result, contemporary research emphasizes the microbiological effects of treatments in addition to their mechanical and clinical usefulness [72]. More accurate characterization of post-treatment microbiota changes has been made possible by sophisticated DNA sequencing and metagenomic techniques, underscoring the significance of customized strategies based on unique microbial profiles [73].

Furthermore, ongoing surveillance of the oral microbiota is necessary for the long-term therapy of periodontitis [74]. It takes routine maintenance procedures and proper at-home dental hygiene to maintain the positive results of initial treatments [75].

In order to improve periodontal health, future research should concentrate on creating targeted medicines that can specifically modify the oral flora [47,76]. Novel techniques, like the application of probiotics and microbiome-based treatments, provide prospects for enhancing periodontitis treatment [77].

In conclusion, the ability of non-surgical and surgical treatments to modify the oral microbiota is critical to the therapy’s long-term efficacy, even if both are necessary for treating periodontitis [78,79,80,81]. A thorough comprehension of these microbiological alterations will aid in the creation of more individualized and efficient treatment plans, thereby enhancing the prognosis for periodontitis patients [82].

### 1.3. The Purpose and Objective of the Review

The goal of this comprehensive review of the literature is to investigate in detail how non-surgical and surgical treatments for periodontitis affect the oral microbiota and microbiome [83]. Microbial factors and the human immune response interact intricately in the chronic inflammatory illness known as periodontitis, which affects the tissues that support the teeth [84]. Since dysbiosis of the oral microbiota contributes significantly to the onset and course of the illness, it is critical to comprehend the ways in which different therapeutic approaches can affect these microbial communities [85].

The objective of this review is to identify the microbiological alterations caused by various therapy modalities by critically analyzing the current findings [86].

It is well recognized that non-surgical procedures like SRP that involve mechanical debridement lower the pathogenic bacterial load and foster a better oral environment [87]. Further research is necessary to determine their long-term impact on the oral microbiota and its capacity to preserve a healthy microbial community [88]. Similarly, oral microbial ecology may be dramatically altered by surgical therapies, which may involve regenerative and resective techniques [89]. We will be able to comprehend the impact of these interventions on the structure and function of the oral microbiota through the analysis of research conducted with the use of metagenomic and sophisticated DNA sequencing techniques [90]. It is essential to assess how these therapies affect the makeup and functioning of the oral microbiota in order to better understand the mechanisms underlying healing as well as to create more specialized and potent treatment plans [91]. To find patterns linked to positive clinical outcomes, variations in microbial diversity, the relative abundance of pathogens and beneficial species, and interactions among microbiota members will be investigated [92].

To sum up, this systematic review will offer a thorough summary of the modifications to the oral microbiota and microbiome brought about by both non-surgical and surgical periodontitis treatments [93,94,95,96,97,98]. We hope to clarify the processes by which these interventions affect oral microbial populations and assess their clinical implications for better periodontitis care and recurrence prevention through a thorough examination of the existing data [99]. Patients with periodontitis will have much better results thanks to this integrated approach, which will aid in the development of more individualized and efficient treatment plans [100].

## 2. Materials and Methods

### 2.1. Protocol and Registration

This review was carried out in accordance with PRISMA (Preferred Reporting Items for Systematic Reviews and Meta-Analyses) guidelines, and it was registered under the number CRD42024553599 on PROSPERO (the International Prospective Register of Systematic Reviews) [101].

### 2.2. Search Process

We limited our search to English-language papers published between 1 January 2004 and 7 May 2024 in PubMed, Scopus, and Web of Science that were relevant to our topic. In the search approach, the Boolean keywords “oral microbio*” AND “periodontitis” were used. We selected these phrases because they most accurately reflected our investigation’s aim, which was to gain additional insight into the interaction between the oral microbiota and periodontitis by identifying the microbiological alterations caused by various therapy modalities through a critical analysis of the current findings (Table 1). 

### 2.3. Inclusion Criteria

Three reviewers evaluated all relevant papers based on the following chosen criteria: (1) solely human subject studies; (2) complete text; and (3) scientific studies evaluating the microbiota’s modifications after periodontal treatment. The following process was used to construct the PICO model:

- Criteria: application in the present study;
- Population: healthy human subjects;
- Intervention: surgical and non-surgical treatment of periodontitis;
- Comparison: control group;
- Outcome: evaluation of microbiota composition after periodontal treatment;
- Study design: clinical trial.

### 2.4. Exclusion Criteria

Articles written in languages other than English, ineligible study designs, ineligible outcome measures, ineligible populations, case studies, reviews, and animal studies were among the exclusion criteria.

### 2.5. Data Processing

Author disagreements on the choice of articles were addressed and settled.

### 2.6. Article Identification Procedure

The appropriateness evaluation was carried out independently by two reviewers, F.I. and F.P. An additional manual search was conducted to increase the number of articles available for full-text analysis. English-language articles that met the inclusion criteria were taken into consideration, and duplicates and items that did not qualify were marked with the reason they were not included.

### 2.7. Study Evaluation

The article data were independently evaluated by the reviewers using a special electronic form designed according to the following categories: authors, year of study, aim of study, materials and methods, and results. The special electronic form used is ROBINSON. This tool was developed to provide a systematic and comprehensive assessment of the risk of bias in studies that do not use randomization to assign participants to intervention groups.

### 2.8. Quality Assessment

Two reviewers, F.P. and P.A., evaluated the included papers’ quality using the ROBINS-I tool. In order to evaluate the possibility of bias in the outcomes of non-randomized trials comparing the health impacts of two or more therapies, ROBINS-I was created. Each of the seven evaluated points was given a bias degree. F.I., the third reviewer, was consulted in case of disagreement until a consensus was reached. The reviewers were instructed on how to use the ROBINS-I tool and adhered to the guidelines in order to assess the potential for bias in seven different domains: confounding, participant selection, intervention classification, deviations from intended interventions, missing data, outcome measurement, and choice of reported results. Discussion and consensus were used to settle any differences or conflicts amongst reviewers in order to improve the assessments’ objectivity and uniformity. In situations when an agreement could not be reached, the final decision was made by a third reviewer. An extensive assessment of potential biases in the non-randomized studies included in this study was made possible by the use of ROBINS-E for bias assessment. It contributed to the overall evaluation of the caliber and dependability of the results by pointing out the evidence base’s advantages and disadvantages. The writers of this review were able to reach more informed interpretations and conclusions based on the facts at hand by taking the risk of bias into account.

## 3. Results and Discussion

**Figure 1:**
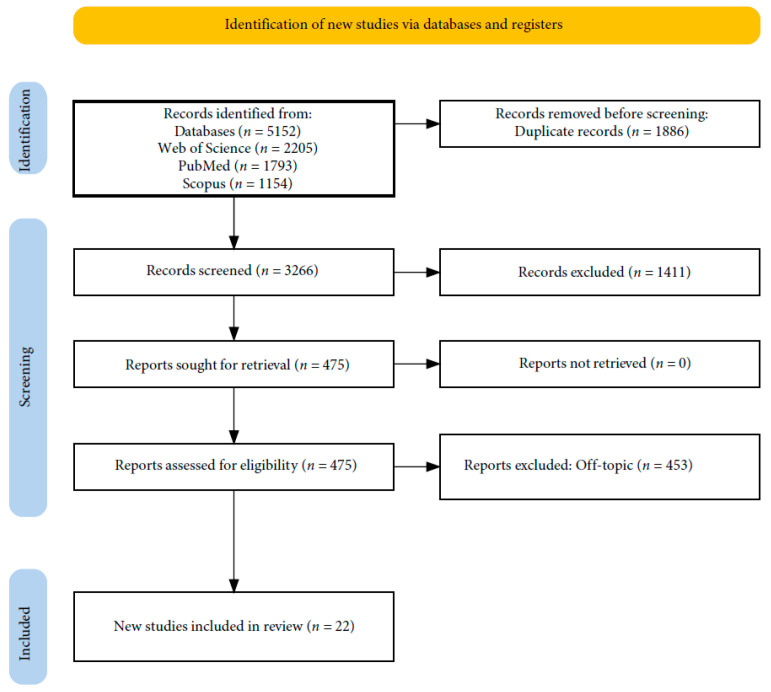
A PRISMA flowchart of the literature search and article inclusion process.

A total of 5152 papers were obtained from the databases Web of Science (2205), PubMed (1793), and Scopus (1154). This resulted in 3266 articles after eliminating duplicates (1886), and 1411 entries were eliminated after their titles and abstracts were examined. The writers were able to successfully obtain the remaining 475 papers and confirm their eligibility. Of these, 453 items were eliminated as a result of this process because they were off-topic. A qualitative analysis of the 22 final articles is included in this study (Figure 1). Each study’s findings are presented in Table 2.

### 3.1. Quality Assessment and Risk of Bias in Included Articles

**Figure 2:**
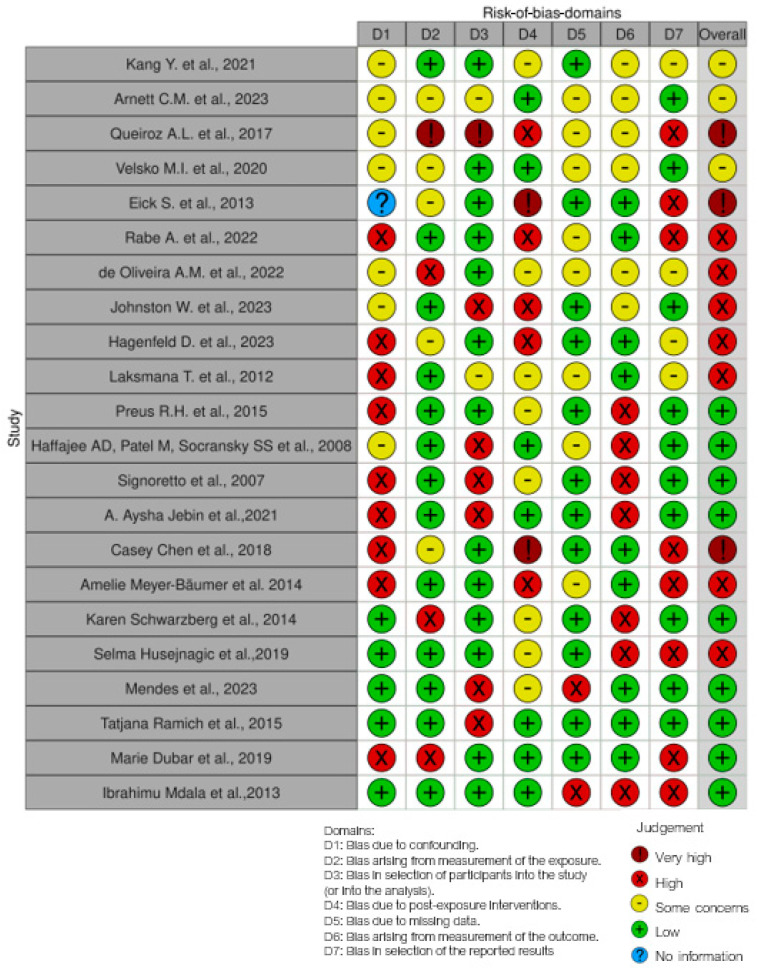
Bias assessment [20,102,103,104,105,106,107,108,109,110,111,112,113,114,115,116,117,118,119,120,121,122].

The risk of bias in the included studies is reported in Figure 2. Regarding the bias due to confounding, most studies have a high risk. The bias arising from measurement is a parameter with a low risk of bias. Many studies have a low risk of bias due to bias in the selection of participants. Bias due to post-exposure could not be calculated due to high heterogeneity. The bias due to missing data is low in many studies. Bias arising from the measurement of the outcome is low. Bias in the selection of the reported results is high in most studies. The final results show that nine studies have a low risk of bias, three have a very high risk of bias, and seven have a high risk of bias.

### 3.2. Discussion

#### 3.2.1. Microbial Dynamics in Periodontal Disease and Treatment Outcomes

The study by Kang et al. examined the consequences of periodontitis and SRP treatment, in addition to the significance of the human oral microbial population as a reservoir for antibiotic resistance [102]. The researchers sought to gain a better understanding of the prevalence of antibiotic- and metal-resistance genes (ARGs, MRGs) by analyzing metagenomic data from 48 DP specimens in the healthy state (HS), 40 in the periodontitis state (PS; before treatment), and 24 in the resolved state (RS; after SRP treatment) [123]. *Campylobacter rectus*, *Tannerella forsythia*, and *Fretibacterium fastidiosum* have all been found to be important bacterial species that significantly influence alterations in the DP microbiota that occur during periodontitis. ARG and MRG numbers increased in response to both periodontitis and SRP therapy, dramatically changing their composition [65]. The ARGs linked to bacitracin resistance were the most prevalent.

Another study also highlighted a coselection phenomenon, where ARGs, MRGs, and mobile genetic elements (MGEs) often co-occurred in the DP microbiota [124]. This suggests complex interactions between microorganisms and resistance genes, which could inform the development of new antimicrobial strategies [125].

Overall, the findings underscore the importance of monitoring ARGs and MRGs in the oral microbiota to improve the efficacy of periodontitis treatments and guide correct antibiotic use. This research provides a foundational understanding that could lead to better therapeutic approaches to managing periodontal disease [126].

The study by Arnett et al. evaluated the effects of SRP alone versus SRP combined with minocycline hydrochloride microspheres (SRP + MM) on periodontal pathogens and clinical outcomes in 70 participants with stage II–IV Grade B periodontitis [103]. Participants were randomized into two groups: 35 received SRP alone, and 35 received SRP + MM. Saliva samples and clinical outcome data were collected at baseline, 1 month, and 3 and 6 months after treatment. The results showed that the SRP + MM group had a significant reduction in several key periodontal pathogens, including *Tannerella forsythia*, *Treponema denticola, Fusobacterium nucleatum*, *Prevotella intermedia*, *Parvimonas micra*, and *Eikenella corrodens*, at 30 days’ evaluation [127]. By 6 months, after reapplying MM at 3 months, there were sustained reductions in *Fusobacterium nucleatum*, *Prevotella intermedia*, *Campylobacter rectus*, and *Eikenella corrodens*. Clinically, the SRP + MM group demonstrated significant improvements, with a reduction in pocket depths of ≥5 mm observed at the 1-month, 3-month, and 6-month evaluations [128]. Additionally, there were notable gains in clinical attachment loss at the 6-month mark. These findings suggest that the addition of minocycline microspheres to SRP not only enhances the reduction in specific periodontal pathogens but also contributes to better clinical outcomes over six months. This combination therapy could be a valuable approach to managing periodontitis more effectively than SRP alone [129].

Velsko et al.’s study investigated the impact of periodontal treatment on the microbiological type in 53 African-American individuals aged 5–25 with Grade C Molar–Incisor Pattern Periodontitis (C/MIP) [105]. Participants were treated with an entire mechanical debridement and systemic antibiotics (MTZ 250 mg + AMX 500 mg, three times daily for 7 days) [130,131,132,133]. Subgingival specimens were collected before treatment and at 3, 6, 12, 18, and 24 months post-therapy and analyzed using a 16S rRNA gene-based microarray [134].

Results showed that treatment effectively reduced clinical disease parameters. A.a. was strongly correlated with untreated sites, while health-associated species included *Rothia dentocariosa/mucilaginosa*, *Eubacterium yurii*, *Parvimonas micra*, *Veillonella* spp., *Selenomonas* spp., and *Streptococcus* spp. [135]. Post-treatment, there was a significant reduction in A.a. and other relevant pathogens and an increase in health-associated species, with these changes maintained for at least 6 months [136].

In conclusion, periodontal therapy significantly reduced disease-associated bacteria, particularly A.a., and shifted the microbial profile toward a healthier state, highlighting its effectiveness in managing C/MIP. Further studies could explore the long-term sustainability of these microbial changes [137].

The randomized placebo-controlled clinical trial by de Oliveira et al. investigated the immediate efficacy of integrated probiotics supplementary to subgingival instrumentation (SI) in patients with untreated periodontitis [108]. Despite significant reductions in subgingival species and clinical improvement in both groups, probiotics did not provide additional benefits. However, a trend for fewer weak subjects in the probiotic group was observed. Significant associations between oral and fecal species were noted, with distinct species associated with poor therapeutic response. Individuals formed groups according to different periodontitis oral–gut bacterial colonies, correlating distinctively with gingival detachment post-therapy [138]. These findings suggest that while systemic probiotics did not enhance short-term periodontal treatment outcomes, they may influence therapeutic response based on individual oral–gut microbial profiles. Further research is warranted to elucidate long-term effects and personalized treatment implications [139].

#### 3.2.2. Therapeutic Approaches and Clinical Implications

Research by Queiroz et al. examined the effect of enamel matrix derivative (EMD) on the periodontal microbiome in 39 patients with mandibular Class II buccal furcation defects [104]. Patients were randomized into three groups: beta-tricalcium-phosphate/hydroxyapatite graft (BONE group), EMD + BONE, and EMD alone. Plaque samples were collected at baseline, 3 months, and half a year after therapy, with 169,000 sequences analyzed against the Human Oral Microbiome Database. Initially, 422 classes were detected, including *Fusobacterium*, *Pseudomonas*, *Streptococcus, Filifactor*, and *Parvimonas* [80]. All treatments modified the disease-associated microbiome, restoring health-compatible species. EMD and EMD + BONE groups showed more significant long-term reductions in species, particularly disease-associated ones like *Selenomonas noxia*, *F. alocis*, and *Fusobacterium*, compared to the BONE group (*p* < 0.05). EMD treatment thus effectively reduces pathogen richness and increases commensal abundance, indicating its potential for improving periodontal health through microbial modulation. Further research is needed to assess how these changes impact periodontal regeneration outcomes [140].

The randomized clinical trial by Eick et al. investigated the impact of hyaluronan-containing gels on early wound healing post-SRP in 34 individuals with chronic periodontitis [106]. Participants were divided into test (*n* = 17) and control (*n* = 17) groups. The results showed significant reductions in probing depth (PD) and clinical attachment level (CAL) in both groups (*p* < 0.001). However, the test group exhibited a greater PD reduction and fewer pockets having PDs ≥ 5 mm at 3 and 6 months (*p* = 0.014/0.021 and *p* = 0.046/0.045, respectively). *Treponema denticola* counts decreased significantly in both groups (*p* = 0.043), while *Campylobacter rectus* counts were reduced significantly only in the study cohort (*p* = 0.028). Notably, *Prevotella intermedia* and *Porphyromonas gingivalis* increased in the placebo cohort. Overall, adjunctive hyaluronan application post-SRP showed promise in enhancing PD reduction and potentially preventing periodontopathogen recolonization. Further research could explore its long-term effects on periodontal health [141].

The pilot study by Rabe et al. explored the impact of lactoperoxidase (LPO) concentrations on initial biofilm accumulation and cellular reactions using a metaproteome approach combined with a model biofilm-regrowth study [107]. Sixteen individuals underwent four local treatments: LPO lozenges in varying concentrations, a control treatment, and Listerine^®^. Mass spectrometry analysis identified 1916 metaproteins per sample, spanning 116 genera and 1316 biomolecular roles. Listerine^®^ reduced metaprotein abundance, confirming its plaque-inhibiting effect. In contrast, LPO lozenges increased the abundance of primary and secondary settlers, including bacteria linked to dental health and periodontitis. A functional analysis suggested plaque biofilm growth [142]. The findings underscore the differential mechanisms of plaque biofilm formation between Listerine^®^ and LPO-containing lozenges, with the latter promoting higher bacterial diversity. Further research could elucidate the clinical implications of these findings on oral health interventions [143].

In Johnston et al.’s investigation of an RCT, 38 periodontitis patients underwent subgingival instrumentation using hand (*n* = 20) or ultrasonic instruments (*n* = 18) [109]. Both groups exhibited comparable biofilm composition at all time points. Large-scale changes were observed within groups, with reduced taxonomic diversity and dysbiosis at days 1 and 7, followed by an increase in health-associated genera. By day 90, some samples had reverted to a microbiome similar to baseline, irrespective of the tool type and remaining illness. These findings suggest that both hand and high-frequency tools have similar consequences for the subgingival biofilm microbiome, with noticeable initial alterations in biofilm composition, although their association with treatment outcomes is limited [144].

In Hagenfeld et al.’s auxiliary evaluation of an RCT involving 163 patients with stage III–IV periodontitis, the impact of adjunctive antibiotics on long-term microbiota changes was investigated [110]. The study administered 500 mg AMX and 400 mg MTZ three times per day regularly for 1 week alongside periodontal therapy. The results revealed significant alterations in 72 ribosomal sequence variants, including notable shifts in key periodontal pathogens such as *Porphyromonas gingivalis* and *Tannerella forsythia* [145]. Moreover, the subgingival microbial dysbiosis index demonstrated a substantial decrease in the pharmacotherapy cohort across all time points, suggesting a favorable shift toward a healthier oral microbiome. While differences in variety between cohorts were not consistently significant at certain intervals, the overall trend observed over 26 months indicates that adjunctive antibiotics contribute to sustained improvements in oral microbiota composition following periodontal therapy [146]. These findings underscore the potential of adjunctive antibiotic therapy in promoting long-term oral health outcomes in patients with advanced periodontitis [147].

In the feasibility study by Laksmana et al., an advanced metagenomic capacity procedure was applied to examine the pre- and post-therapy subgingival plaque in subjects diagnosed with AgP [111]. Through DNA extraction, PCR amplification, and sequencing, a total of 24,673 reads were obtained, identifying 208 species/phylotypes. Notably, 129 of these species/phylotypes were found in both patients, although their relative proportions varied [148]. Interestingly, each sample contained over 120 species/phylotypes, with 28–42 species/phylotypes collectively representing 90% of subgingival bacteria [149]. Post-treatment analysis revealed alterations in the subgingival microbial community composition, suggesting a dynamic response to therapy. These findings underscore the high species richness present in the subgingival microbiota, with a few dominant species/phylotypes influencing the overall microbial diversity. Overall, this study demonstrates the feasibility and utility of utilizing advanced metagenomic capacity evaluation to explore the intricate dynamics of subgingival microbiota in periodontitis patients at the beginning and at the end of therapeutic interventions [150].

In Preus et al.’s RCT involving 184 patients with chronic destructive periodontitis, the connection between therapeutic and bacterial results was investigated across four treatment strategies [112]. Patients were assigned to receive either full-mouth disinfection (FDIS) or SRP, with or without additional MTZ [151]. Initial microbiologic assessments revealed relatively low numbers of microbial varieties [152]. After 12 months, a reduction in *Tannerella forsythia* emerged as the sole microbial factor significantly associated with clinical outcomes, particularly the lack of PDs ≥ 5 mm. This finding underscores the potential of *T. forsythia* as a promising marker for treatment effectiveness in chronic periodontitis. Further research may elucidate its utility in guiding therapeutic interventions and improving long-term patient outcomes [153].

#### 3.2.3. Microbiological Changes and Therapies for Chronic Periodontitis

In a study by Haffajee et al., 92 participants with chronic periodontitis were treated with four different approaches: “SRP alone, SRP combined with azithromycin, SRP combined with metronidazole, and SRP combined with doxycycline” [20,154]. Clinical changes were observed one year post-therapy, with SRP performed under local anesthesia and adjuvant antimicrobials initiated at the first visit. Maintenance included SRP at monitoring appointments to ensure continued periodontal health [155]. All treatments significantly reduced pathogenic bacteria, particularly red and orange complexes, improved the attachment level, and reduced the pocket depth [156,157]. The study emphasized the importance of prompt bacterial reductions for effective treatment and long-term stability. While antibiotic-resistant isolates increased during treatment with medication, they had returned to baseline by 12 months [158]. Combination therapies typically yielded better results, highlighting the importance of maintaining microbial balance to prevent harmful organism resurgence. Using antibiotics as adjuncts to mechanical debridement underscores the importance of achieving rapid and substantial bacterial reductions for long-term periodontal health [159,160].

Casey Chen et al. revealed significant differences in microbial communities between dental plaque and saliva, as well as between periodontally healthy and diseased sites [115,161,162]. Using samples from 21 individuals with healthy gums and 48 adults with chronic periodontitis, an examination and microbiological sampling were conducted, showing substantial inter-individual heterogeneity in post-treatment microbiome changes [163]. Disease-associated bacteria thrived in conditions conducive to health, with changes in the microbiome resulting from proportionate increases in harmful bacteria.

The stability of microbiota differed between subgingival plaque and saliva, with varied roles of dispersal mechanisms in community turnover [118,164]. After treatment, saliva exhibited a greater number of variables, indicating widened oral habitats, while dental environments post-treatment were less uniform, influenced by stochastic mechanisms [165].

Meyer-Bäumer et al.’s study aimed to assess factors contributing to disease recurrence in patients with aggressive periodontitis (AgP) who underwent post-active periodontal therapy (APT) [116,166]. Microbiological samples from 73 AgP patients revealed notable reductions in key periodontal pathogens following non-supportive periodontal therapy (NSPT). Adjuvant antibiotic therapy significantly decreased A.a. levels, supporting its role in long-term periodontal therapy [167,168].

The recurrence of periodontal disease at reexamination was associated with increased *Tannerella forsythia* and *Treponema denticola* levels, particularly in smokers. Smoking was identified as a major risk factor, correlating with higher levels of these pathogens [169]. The study underscores the importance of continued monitoring and personalized periodontal care, especially for high-risk individuals like smokers [116].

Another study evaluated the microbiological effects of combining SRP with antimicrobial chemical control in periodontitis treatment [170]. Sixty participants received either SRP alone or SRP plus metronidazole (MTZ) + amoxicillin (AMX) for 14 days, with or without chlorhexidine mouthwash (CHX) for 60 days. Microbiological samples collected up to 180 days post-treatment revealed variations in microbial patterns across oral cavity regions [46,171]. Untreated bacteria in saliva, serving as a reservoir of microorganisms, can potentially recolonize the subgingival region if they spread from saliva or soft tissues [172]. Therefore, reducing saliva-borne pathogens is crucial for preventing subgingival reinfection. Antibiotics, in conjunction with other oral habits, synergistically reduce subgingival infection, providing insight into the clinical and microbiological impacts of periodontal therapy on subgingival recolonization [114,173].

Combining systemic and local antimicrobial chemical control with basic periodontal therapy significantly impacts the oral microbial composition, particularly in subgingival biofilm and saliva [174,175]. Extra-crevicular sites exhibit distinct microbial patterns compared to intra-crevicular sites (subgingival plaque), and combined antimicrobial chemical control minimizes subgingival recolonization around remaining periodontal pockets [176].

In another study, 30 patients with either generalized severe chronic (*n* = 18) or aggressive (*n* = 12) periodontitis were examined. Before the combined antibiotic and mechanical treatment (AT), A.a. was found subgingivally in all cases [177]. Following AT, subgingival plaque samples were collected from the four deepest sites (DEEP) and the original four sites (ASPRE) [178]. The detection rate of A.a. decreased from 100% to 0% (ASPRE) or 7% (DEEP) after combined mechanical–antibiotic therapy. The difference between ASPRE and DEEP detection rates was not statistically significant (*p* = 0.157), possibly due to the modest sample size [179,180].

After treatment, *T. forsythia* detection was 27% (ASPRE) and 53% (DEEP), while *P. gingivalis* and *T. denticola* were detected at rates of 47–53%. Red complex bacteria were more effectively suppressed by the combined therapy compared to A.a. [181]. Although there was no significant difference in *P. gingivalis* and *T. denticola* detection rates between ASPRE and DEEP, *T. forsythia* was more frequently (*p* = 0.005) and abundantly (*p* = 0.001) detected using DEEP sampling. Thus, DEEP sampling may be preferred post-treatment. However, the combined therapy reduced A.a. detection below 10% but was unable to suppress *P. gingivalis*, *T. forsythia*, and *T. denticola* detection rates below the limit [119,182].

Mdala et al. observed changes in subgingival bacterial counts in deeper pockets (≥5 mm) following periodontal treatments over two years [122,183]. They studied eight treatment modalities and analyzed samples from 176 participants using checkerboard hybridization [184]. Short-term reductions in red complex bacteria were seen with AMOXMETTET therapy, but long-term effects were minimal, suggesting that early microbial suppression does not guarantee long-term periodontal health. Smoking significantly undermines therapy efficacy, increasing pathogenic bacteria colonization, especially *P. gingivalis* [185,186]. Consistent maintenance therapy and strict oral hygiene are crucial for preventing periodontal disease recurrence. Despite various treatments, there were no significant long-term microbiome alterations [187].

#### 3.2.4. Microbiota and Advanced Treatments in Periodontal Therapy

Schwarzberg et al. examined 36 individuals, aged 21 to 40, from an American Indian/Alaska Native (AIAN) community, including patients with gingivitis, mild-to-moderate periodontitis, severe periodontitis, and healthy controls [117,188]. Periodontal disease is prevalent in the AIAN community, making such studies essential. PD, CAL, plaque scores, and BOP were measured to assess disease severity, with follow-up visits conducted after periodontal therapy completion [182,183,184,185,186,187,188,189]. The analysis of 76 periodontal pocket microbial population samples using 454 pyrosequencing revealed that treatment, including SRP, did not significantly alter the oral microbiome’s overall composition [190,191]. Despite clinical improvements, the microbiome remained consistent with pre-treatment states, indicating dominant individual-specific microbiome characteristics. This complicates efforts to identify universal microbial markers for disease progression or recovery, although specific bacterial changes were noted [192].

Signoretto et al. aimed to compare the effects of hyperbaric oxygen (HBO) therapy versus surgical intervention (SRP) and their combination on periodontal pocket microflora changes in adult patients with chronic periodontitis [113,193]. Twelve female patients, aged 30–45, with periodontal pockets > 6 mm were selected from the University of Verona Dental Clinic. The study divided the oral cavity into two halves: one underwent SRP surgery, while the other received no therapy. Ten patients received a 10-day HBO cycle of ten sessions each. Four sites were analyzed: (1) pockets treated with SRP and HBO together; (2) pockets treated with HBO alone; (3) pockets treated with SRP alone; (4) untreated pockets [194]. Microbiological data revealed that both SRP and HBO alone temporarily reduced the Gram-negative anaerobe load, but the effects were not sustained, reverting to baseline within a month. Conversely, combining SRP and HBO led to a profound and sustained reduction in anaerobe load, remaining significantly lower than baseline even three months post-treatment [120,195]. The study suggests better microbiological and clinical outcomes with HBO, especially in conjunction with SRP, effectively lowering pathogenic bacterial loads and sustaining gingival health over time [196].

In the study by Jebin et al., 24 males and 6 females with chronic periodontitis (CP), aged 20 to 60, were included [114,197]. Patients were randomly assigned to two groups: one received SRP and a daily probiotic tablet containing *L. reuteri* UBLRu-87 for a month; the other received SRP alone. Clinical and microbiological indicators were assessed at baseline, 1 month, and 3 months [198]. Both groups showed decreased Pg levels from baseline to three months, with the test group experiencing more significant reductions (*p* ≤ 0.05). Additionally, the test group showed a statistically significant increase in *L. reuteri* levels, indicating effective colonization and potential long-term benefits for periodontal health [199,200]. At the three-month evaluation, the test group required less surgical intervention than the control group, with a significantly larger reduction in locations requiring surgery. Probiotic therapy in addition to SRP improves clinical outcomes and reduces the need for invasive procedures in chronic periodontitis treatment [201,202]. This approach offers microbiological and clinical advantages over SRP alone, highlighting probiotics’ potential to enhance periodontal therapy outcomes [114,203].

In their study, Husejnagic et al. investigated the clinical and microbiological effects of additional photoactivated disinfection (PAD) in periodontitis treatment using a red LED light source [118,204,205]. Twenty participants with periodontitis were included in this split-mouth study and randomly assigned to the test or control group for each side of the jaw. Following conservative periodontal therapy, the test group received two sessions of adjunctive photo-biomodulation therapy (PAD) using a red LED at 635 nm and 0.01% tolonium chloride, while the control group did not receive PAD. Clinical periodontal examination criteria, including PD, CAL, BOP, and microbiological assays (PCR), were assessed before and after treatment [206,207]. Both groups showed improvements in bleeding values, attachment gain, and PD reduction, with no statistically significant extra advantage seen in the group treated with PAD with red LED compared to debridement alone. Although there appeared to be less recolonization of *P. gingivalis* and *T. denticola* after adjuvant therapy with a red LED, larger sample sizes are needed for confirmation. Overall, adjuvant PAD with a red LED did not provide additional therapeutic benefits over debridement alone in chronic periodontitis cases [117,208].

In 2019, Dubar et al. evaluated the subgingival microbiota of 30 periodontitis patients [115,121,122,209,210,211,212,213]. They collected samples from diseased and healthy sites before and after SRP, alongside data on clinical variables [210,211]. Protozoa, particularly *E. gingivalis* ST1, were frequently found in periodontitis patients, correlating strongly with gingival inflammation and CAL, especially in deeper pockets. *E. gingivalis* ST1 was associated with a high bacterial load, suggesting a symbiotic relationship with periodontal pathogens. *T. tenax*, also identified, correlated with other periodontal infections but decreased after non-surgical treatment, indicating susceptibility to traditional therapies [122,212]. However, protozoa presence did not significantly correlate with treatment outcomes, suggesting that they may act as reservoirs for pathogens, influencing periodontal disease evolution indirectly [213].

In order to maintain tissue homeostasis and combat pathogenic assaults, neutrophils are essential. The study by Bassani et al. examined their involvement in the development of systemic comorbidities and the advancement of periodontitis, a chronic inflammatory disease that affects the tissues that support the teeth. Oral microbiota dysbiosis is the cause of periodontitis, which results in tissue damage and persistent gingival inflammation. As one of the first responses to infection, neutrophils release antimicrobial compounds but, if stimulated repeatedly, can also induce tissue damage and bone resorption. Because of this ongoing inflammation, systemic disorders like diabetes, rheumatoid arthritis, and cardiovascular problems are linked to periodontitis. Comprehending how neutrophil malfunction affects systemic health and periodontitis is essential to creating treatments that inhibit their activation, enhancing patient care and illness control [214].

The observational study by Pardo et al. examined the relationship between oral infections and periodontal disease in specimens from 26 patients having aortic valve replacement surgery. After undergoing soft tissue tests, 19 individuals had 3 cases of severe periodontitis and 12 cases of mild periodontitis. Aortic valve specimens, dental plaque or saliva samples, and 16S rRNA gene sequencing were used in the analysis process. DNA from periodontopathic and oral bacteria was found in nine valve samples, indicating that these pathogens may have moved from the oral cavity to the heart tissue. The significance of periodontal health in cardiovascular disease is shown by the non-coincidental presence of mouth infections in valve tissue. These results emphasize the necessity of additional studies to comprehend the causes and effects of bacterial translocation in order to enhance patient care and prevention for those who have periodontal and cardiovascular diseases [215].

### 3.3. Limitations of the Study

Research on periodontal disease and treatment strategies reveals significant insights into the complex interplay of oral microbiota and the efficacy of various interventions. Periodontitis significantly alters the oral microbiome, increasing the prevalence of antibiotic- and metal-resistance genes, with certain bacterial species playing key roles in these changes. Both periodontitis and SRP increase antibiotic-resistance genes, suggesting intricate microbial interactions that can inform new antimicrobial strategies. Monitoring resistance genes in oral microbiota is crucial for optimizing treatments. Combining SRP with minocycline hydrochloride microspheres significantly reduces key periodontal pathogens and improves clinical outcomes, sustaining reductions in harmful bacteria and enhancing clinical attachment. Comprehensive mechanical debridement and systemic antibiotics effectively reduce disease-associated bacteria and promote health-associated species, maintaining positive changes for at least six months. The impact of probiotics as an adjunct to subgingival instrumentation reveals potential long-term benefits based on individual oral–gut microbial profiles, underscoring the need for personalized treatment approaches. EMD, especially when combined with a bone graft, significantly reduces disease-associated species and promotes a healthier microbial balance, highlighting its potential in periodontal regeneration. Hyaluronan-containing gels enhance early wound healing post-SRP, significantly reducing pocket depths over six months. LPO lozenges may promote bacterial diversity, while Listerine^®^ effectively reduces plaque biofilm. Both hand and ultrasonic instruments for S.I. show similar biofilm composition changes and clinical outcomes, indicating their equivalence. Adjunctive antibiotics in periodontal therapy lead to sustained microbiota improvements, reducing key periodontal pathogens and supporting long-term oral health. Advanced metagenomic techniques reveal dynamic microbial community changes in response to therapy, highlighting the complexity of the oral microbiome. Specific bacterial markers, such as Tannerella forsythia, can guide treatment effectiveness. Combining mechanical debridement with antimicrobial agents improves clinical parameters, although the microbiome composition may remain consistent with pre-treatment states, indicating the need for targeted microbial markers. Hyperbaric oxygen therapy with SRP shows the potential to improve periodontal therapy outcomes. Probiotics and photoactivated disinfection as adjunctive therapies to SRP show varied benefits, with probiotics reducing pathogenic bacteria and improving clinical outcomes, while photoactivated disinfection does not provide significant additional benefits. Overall, the research underscores the multifaceted nature of periodontal disease and the importance of tailored therapeutic approaches. Integrating advanced microbial analyses and personalized treatment strategies holds promise for improving periodontal health and managing antibiotic resistance in oral microbiota.

### 3.4. Future Directions

Below are some of the possible research directions in this field.

Personalized Therapies: Develop individualized treatments using advanced sequencing to create microbial profiles. Investigate the use of probiotics and microbiome-based treatments to enhance periodontitis management and restore oral health.

Ongoing Monitoring: Implement continuous monitoring of oral microbiota through wearable biosensors or regular assessments to maintain treatment effectiveness and prevent recurrence. Conduct long-term studies to assess the sustainability of treatment outcomes and the overall health of the oral microbiome.

Mechanistic Understanding: Examine the effects of different treatments on oral microbial populations and their role in the healing process. Correlate changes in microbial diversity and pathogen levels with clinical outcomes to understand the direct impact on periodontitis.

Innovative Treatment Approaches: Combine traditional treatments with adjunctive therapies, such as antibiotics and probiotics, to improve effectiveness and reduce recurrence. Employ advanced technologies like metagenomics and metabolomics to gain insights into oral microbiota dynamics during treatment.

These objectives aim to create more effective and personalized treatments for periodontitis, enhancing patient care and advancing research in periodontal health.

## 4. Conclusions

Research on periodontal disease highlights the complex relationship between the oral microbiome and various treatments. Periodontitis increases antibiotic- and metal-resistance genes, necessitating new antimicrobial strategies and regular monitoring of these genes. Combining SRP with minocycline microspheres improves outcomes by reducing key pathogens. Proactive measures should include comprehensive mechanical debridement with systemic antibiotics to sustain positive changes for at least six months. Emphasizing personalized treatments, the integration of probiotics, EMD with bone grafts, and hyaluronan-containing gels shows promising long-term benefits. Additionally, the use of LPO lozenges and Listerine^®^ can enhance bacterial diversity and reduce plaque biofilm, with hand and ultrasonic instruments proving equally effective for subgingival instrumentation. Adjunctive antibiotics improve long-term oral health, and advanced metagenomic techniques reveal microbial changes post-therapy, aiding treatment strategies. Combining mechanical debridement with antimicrobials improves clinical parameters, while hyperbaric oxygen therapy shows potential benefits. Probiotics reduce pathogenic bacteria, but photoactivated disinfection adds little benefit. Overall, personalized, advanced microbial analyses and treatment strategies promise better periodontal health and antibiotic resistance management.

## Figures and Tables

**Table 1:** Indicators for database searches.

Article-screening strategy	KEYWORDS: “A”: oral microbio*; “B”: periodontitis
	Boolean Indicators: “A” AND “B”
	Timespan: 1 January 2004 to 7 May 2024
	Electronic databases: PubMed; Scopus; Web of Science.

**Table 2:** A descriptive item selection summary.

Authors and Years	Type of the Study	Aim of the Study	Material and Methods	Time of Treatment	Time of Recovery/Follow-Up	Results
Kang Y. et al., 2021 [102]	Cross-sectional study	To characterize the changes in the abundance and composition of ARGs and MRGs in the dental plaque microbiota across different periodontal health states: healthy state (HS), periodontitis state (PS; before treatment), and resolved state (RS; after SRP treatment).	- 48 samples in a healthy state; - 40 samples in a periodontitis state; - 24 samples in a resolved state; - Data selection; - Netshift analysis; - ARG and MRG analysis; - Co-occurrence analysis.	SRP was performed on individuals in the periodontitis state (PS).	Samples for the resolved state (RS) collected after SRP treatment.	- Key microbial drivers; - ARGs and MRGs increase; - Composition changes; - Predominant ARGs; - Predominant MRGs; - Coselection phenomenon.
Arnett C.M. et al., 2023 [103]	RCT	To test the effects of SRP alone versus SRP + MM (SRP + MM) on 11 periodontal pathogens and clinical outcomes in participants with stage II–IV Grade B periodontitis.	Seventy participants with stage II–IV Grade B periodontitis were randomized into two groups: SRP alone (*n* = 35) and SRP + MM (*n* = 35). Saliva and clinical outcomes were collected at baseline before SRP, at 1-month reevaluation, and at 3- and 6-month periodontal recall.	SRP: SRP was performed at the beginning of the study. SRP + MM (minocycline hydrochloride microspheres): MM was applied immediately after SRP and again immediately after the 3-month follow-up for pockets ≥ 5 mm.	1-month reevaluation: Clinical outcomes and saliva samples collected. 3-month follow-up: Periodontal maintenance and reapplication of MM in the SRP + MM group. 6-month follow-up: Final clinical outcomes and saliva samples collected.	There were significant reductions in Tannerella forsythia, Treponema denticola, *Fusobacterium nucleatum*, *Prevotella intermedia*, *Parvimonas micra*, and *Eikenella corrodens* at the 1-month reevaluation after SRP + MM. At 6 months, with the reapplication of MM at 3 months, significant reductions were noted in *Fusobacterium nucleatum*, *Prevotella intermedia*, *Campylobacter rectus*, and *Eikenella corrodens*.
Queiroz A.L. et al., 2017 [104]	RCT	To identify changes in the periodontal microbiome after treatment with EMD using a deep-sequencing approach.	Thirty-nine patients with mandibular Class II buccal furcation defects were randomized into three groups: beta-tricalcium-phosphate/hydroxyapatite graft (BONE group), EMD + BONE group, and EMD-alone group.	Patients received one of three treatments: BONE (beta-tricalcium-phosphate/hydroxyapatite graft), EMD + BONE, or EMD alone.	Baseline: Plaque samples collected before treatment. 3 months post-treatment: Plaque samples collected for analysis. 6 months post-treatment: Final plaque samples collected for analysis.	From the 39 defects, 422 species were identified belonging to genera such as *Fusobacterium*, *Pseudomonas*, *Streptococcus*, *Filifactor* and *Parvimonas*. All three treatments altered the disease-associated microbiome, restoring health-compatible species.
Velsko M.I. et al., 2020 [105]	Longitudinal cohort study	To evaluate the influence of periodontal therapy on the microbiological profiles of individuals with C/MIP.	Fifty-three African-American individuals aged 5–25 diagnosed with C/MIP underwent full-mouth mechanical debridement combined with systemic antibiotics (MTZ250 mg and AMX500 mg, three times a day for 7 days). Subgingival samples were collected from diseased and healthy sites before treatment and at 3, 6, 12, 18, and 24 months post-therapy.	Patients underwent full-mouth mechanical debridement. Systemic antibiotics: metronidazole (250 mg) + amoxicillin (500 mg) three times daily for 7 days.	Subgingival samples collected before treatment. Samples collected again at 3, 6, 12, 18, and 24 months post-treatment.	Treatment effectively reduced the main clinical parameters of the disease. *Aggregatibacter actinomycetemcomitans* (A.a.) was the strongest species associated with diseased sites. Other disease-associated species included *Treponema lecithinolyticum* and *Tannerella forsythia*.
Eick S. et al., 2013 [106]	RCT	To determine the effects of hyaluronan-containing gels on clinical variables, subgingival bacteria, and the local immune response during early wound healing after SRP.	Thirty-four individuals with chronic periodontitis were included. Test group (*n* = 17): received hyaluronan gels with two molecular weights during the first 2 weeks post-SRP. Control group (*n* = 17): received SRP only.	Full-mouth SRP was performed for both groups. Test group: additional application of hyaluronan gels during the first 2 weeks after SRP.	Baseline: Initial measurements and sample collection before SRP. 3 months post-treatment: Measurements and sample collection. 6 months post-treatment: Final measurements and sample collection.	Both groups showed significant reductions in PD and CAL (*p* < 0.001). The test group had significantly greater reductions in PD and fewer pockets with PD ≥ 5 mm at 3 months (*p* = 0.014 and 0.021) and 6 months (*p* = 0.046 and 0.045). *Treponema denticola* counts were significantly reduced in both groups (*p* = 0.043).
Rabe A. et al., 2022 [107]	Randomized cross-over study	To investigate the impact of different concentrations of lactoperoxidase (LPO) on early plaque formation and active biological processes using a metaproteome approach combined with a standard plaque-regrowth study.	Sixteen orally healthy subjects received four local treatments: two lozenges containing components of the LPO system in different concentrations, a placebo, and Listerine^®^. The newly formed dental plaque was analyzed using mass spectrometry (nLC-MS/MS) to identify metaproteins.	Participants received four local treatments with different concentrations of LPO lozenges, placebo, and Listerine^®^ in a randomized, single-blind, cross-over design.	Standard plaque-regrowth study: Dental plaque formation monitored. Mass spectrometry analysis: Newly formed dental plaque analyzed by nLC-MS/MS to identify metaproteins and their functions.	On average, 1916 metaproteins per sample were identified, representing 116 genera and 1316 protein functions. Listerine^®^ reduced the number and relative abundance of metaproteins, confirming its plaque-inhibiting effect. The LPO lozenges mainly increased the abundance of early and secondary colonizers.
de Oliveira A.M. et al., 2022 [108]	Randomized clinical trial	To evaluate the short-term efficacy of systemic probiotics as adjunctive therapy to subgingival instrumentation (SI) in restoring oral–gut microbiotas and improving periodontal clinical outcomes in patients with untreated periodontitis.	Systemically healthy adults with untreated periodontitis were recruited and randomized to receive either SI plus placebo or SI plus probiotics for 30 days. Subgingival biofilm and stool samples were collected at baseline and 2 months post-therapy for microbiological analyses using checkerboard and 16S rRNA gene sequencing.	All participants were treated with SI and took a daily capsule (probiotics or placebo) for 30 days.	Baseline: Subgingival biofilm and stool samples collected before treatment. 2 months post-therapy: Subgingival biofilm and stool samples collected for microbiological analyses.	Most subgingival species and α-diversity decreased significantly (*p* < 0.05), while gut composition and diversity were minimally affected. Both groups showed significant clinical improvement (*p* < 0.05), with a trend for fewer poor responders in the probiotic group (31.5%) compared to the placebo group (60.8%) (*p* = 0.07). Strong correlations between oral and fecal species were observed (*p* < 0.01), and distinct species were associated with poor responses to different therapies (*p* < 0.05).
Johnston W. et al., 2023 [109]	RCT	To investigate early and later changes in the subgingival biofilm following periodontal treatment, assess whether these changes are associated with treatment outcomes, and determine whether the biofilm responds differently to hand versus ultrasonic instruments.	Thirty-eight periodontitis patients underwent full-mouth S.I. using either hand (*n* = 20) or ultrasonic instruments (*n* = 18). Gingival plaque samples were collected at baseline, 1 day, 7 days, and 90 days post-treatment. Bacterial DNA was analyzed using 16S rRNA sequencing. Periodontal clinical parameters were assessed before and after treatment.	Full-mouth S.I. was performed using either hand instruments (*n* = 20) or ultrasonic instruments (*n* = 18).	Baseline: Subgingival plaque sampled before treatment. Day 1 post-treatment: Subgingival plaque sampled. Day 7 post-treatment: Subgingival plaque sampled. Day 90 post-treatment: Subgingival plaque sampled for final analysis.	Biofilm composition was similar in both treatment groups at all time points. Large-scale changes were observed within groups over time, with reduced taxonomic diversity and dysbiosis at days 1 and 7, accompanied by an increase in health-associated genera. By day 90, a subset of samples had reverted to a microbiome comparable to baseline, independent of instrumentation choice and residual disease.
Hagenfeld D. et al., 2023 [110]	RCT	To explore whether adjunctive antibiotics can significantly influence long-term microbiota changes in patients with stage III–IV periodontitis.	The study involved periodontal therapy with adjunctive 500 mg AMX and 400 mg MTZ or placebo thrice daily for 7 days. Subgingival plaque samples were collected before and at 2, 8, 14, and 26 months after mechanical therapy. The V4-hypervariable region of the 16S rRNA gene was sequenced using Illumina MiSeq.	Mechanical periodontal therapy was administered combined with either adjunctive antibiotics (500 mg amoxicillin and 400 mg metronidazole thrice daily for 7 days) or placebo.	Baseline: Subgingival plaque samples collected before treatment. 2 months post-treatment: Subgingival plaque samples collected. 8 months post-treatment: Subgingival plaque samples collected. 14 months post-treatment: Subgingival plaque samples collected. 26 months post-treatment: Subgingival plaque samples collected.	Out of 163 patients with stage III–IV periodontitis, 72 RSVs changed significantly over 26 months due to adjunctive systemic antibiotics, including *Porphyromonas gingivalis*, *Tannerella forsythia*, and A.a. SMDI decreased significantly more in the antibiotic group at all time points. Differences in alpha and beta diversity between groups were not significant at 2, 8, and 14 months.
Laksmana T. et al., 2012 [111]	Feasibility study	To test the feasibility of a high-throughput metagenomic approach for analyzing pre- and post-treatment subgingival plaque in subjects with aggressive periodontitis.	DNA was extracted from subgingival samples and subjected to PCR amplification of the c2–c4 regions of the 16S rDNA using barcoded primers. The PCR products were pooled and sequenced for the v4 region of the 16S rDNA using the 454 FLX standard platform. The results were analyzed for species/phylotypes against the Human Oral Microbiome Database (HOMD) and Ribosomal Database Project (RDP) database.	Periodontal therapy was administered to two subjects with aggressive periodontitis. DNA was extracted from subgingival samples before and after treatment.	Baseline: Subgingival plaque samples collected before treatment. Post-treatment: Subgingival plaque samples collected after treatment.	Sequencing yielded 24,673 reads, identifying 208 species/phylotypes. While 129 species/phylotypes were common in both patients, over 120 species/phylotypes were found in all samples, with 28–42 species/phylotypes collectively representing 90% of all subgingival bacteria in each sample. The remaining species/phylotypes each constituted ≤0.2% of the total subgingival bacteria.
Preus R.H. et al., 2015 [112]	RCT	To investigate the relationship between the clinical and microbiologic outcomes of 4 different treatment strategies for chronic destructive periodontitis among patients maintaining excellent oral hygiene and low gingival bleeding scores.	One hundred eighty-four periodontitis patients capable of maintaining good oral hygiene were randomly assigned to four treatment groups: (1) FDIS + metronidazole; (2) FDIS + placebo; (3) SRP + metronidazole; and (4) SRP + placebo.	Patients were allocated to one of four treatment groups: Full-mouth disinfection (FDIS) + metronidazole; FDIS + placebo; SRP + metronidazole; SRP + placebo.	Baseline: Initial recordings of plaque, bleeding on probing, probing depth (PD), and clinical attachment level. 3 months post-treatment: Follow-up recordings and subgingival samples collected. 12 months post-treatment: Final follow-up recordings and subgingival samples collected.	Baseline bacterial species levels were relatively low. After 12 months, the reduction in Tannerella forsythia was the only microbial factor significantly associated with clinical outcomes, particularly with being free from PD ≥ 5 mm.
Haffajee AD, Patel M, Socransky SS (2008) [20]	Randomized controlled trial	To examine subgingival microbiological changes in chronic periodontitis treated with SRP alone or with azithromycin, metronidazole, or low-dose doxycycline.	Ninety-two subjects with periodontitis were treated with SRP alone or combined with antibiotics. Subgingival plaque samples were taken at baseline, 2 weeks and 3, 6, and 12 months and analyzed for 40 bacterial species using DNA–DNA hybridization.	Patients were treated with a single SRP session alone or in combination with azithromycin administered systemically at a dose of 500 mg once daily for 3 days, metronidazole administered systemically at a dose of 250 mg three times a day (tid) for 14 days, or 20 mg doxycycline (SDD, Periostat^®^; CollaGenex Pharmaceuticals, Newtown, PA, USA) twice daily for 12 weeks.	12 months of follow-up.	All treatments reduced red complex species at 12 months. Antibiotics had significantly reduced red complex species by 2 weeks. Resistant isolates increased during treatment but had returned to baseline by 12 months.
Caterina Signoretto (2007) [113]	Comparative study	To evaluate the effects of hyperbaric oxygen (HBO) on chronic periodontitis compared to SRP and combined therapy.	Twenty patients with chronic periodontitis were treated with HBO, SRP, or both. Bacterial detection was performed using culture and PCR methods.	One session of supragingival oral hygiene by ultrasonic application was administered.	From the collection of two plaque samples for each patient to 75 days after.	The combination of HBO and SRP reduced Gram-negative anaerobes by up to 99.9%, with effects lasting at least two months. HBO or SRP alone had a more limited effect. A significant reduction in periodontopathogens and improved gingival health were observed.
A. Aysha Jebin (2021) [114]	Randomized controlled trial	To evaluate the effects of probiotic chewable tablets with Lactobacillus reuteri on chronic periodontitis.	Thirty CP patients were treated with SRP, divided into two groups: SRP + probiotic tablets and SRP alone. Evaluations were performed at baseline, 1 month, and 3 months.	A chewable probiotic pill was taken once daily (in the evening) following brushing for one month following the first SRP, while the control group only received SRP.	3 months of follow-up.	Significant improvements in clinical and microbiological parameters in the probiotic group compared to SRP alone were observed.
Casey Chen et al. (2018) [115]	Observational study	To examine microbial diversity and community assembly in periodontal health and disease and post-treatment.	Two hundred thirty-eight saliva and subgingival samples from 21 healthy subjects and 48 subjects with periodontal disease were analyzed using 16S rRNA gene sequencing.	Not present	Evaluations started 4 weeks after the non-surgical treatment.	Disease- and health-associated taxa were identified; post-treatment shifts in taxa and community modularity were observed, influenced by ecological drift, dispersal limitation, and homogeneous selection.
Amelie Meyer-Bäumer et al. (2014) [116]	Comparative study	To assess the association between periodontal pathogens and the recurrence of disease in patients with aggressive periodontitis after therapy.	Microbiological samples were taken from 73 patients 5–17 years post-therapy; real-time PCR was used to detect pathogens; uni- and multivariate analyses evaluated associations with recurrence, smoking, and antibiotic therapy.	Forty-two patients (57.5%) received supplementary antibiotics after subgingival debridement was completed during APT. These patients received amoxicillin 375 mg and metronidazole 250 mg 39/day for seven days.	5–17 years	*T. forsythia* and *T. denticola* were significantly associated with disease recurrence; *T. denticola* was also linked to increased clinical attachment levels (CALs) ≥ 6 mm.
Karen Schwarzberg et al. (2014) [117]	Clinical trial	To investigate changes in periodontal pocket bacterial diversity after standard periodontal treatment.	Next-Generation Sequencing was used to characterize bacterial diversity pre- and post-treatment; phylogenetic analysis of periodontal pathogens was performed.	Not present	Patients returned for a follow-up visit at least six weeks after finishing periodontal therapy.	Despite changes in certain taxa post-treatment, samples retained high similarity to pre-treatment samples from the same individual. Unexpected diversity and differential treatment responses among species were noted in *Prevotella* and *Fusobacterium genera*.
Selma Husejnagic et al. (2019) [118]	Randomized controlled trial	To evaluate adjunctive photoactivated disinfection (PAD) in periodontal treatment.	Twenty patients with periodontitis underwent PAD on one side of the jaw, with clinical and microbiological assessments before and after treatment.	After an oral hygiene session, patients underwent 2 to 4 sessions of debridement using manual and ultrasonic instruments.	Final evaluations 3 months after treatment.	Both groups showed significant clinical improvement after 3 months; PAD did not significantly affect bacterial recolonization.
Mendes et al. (2023) [119]	Observational study	To assess the microbiological effects of periodontal therapy with systemic antibiotics and chlorhexidine.	Sixty subjects underwent SRP alone or with antibiotics and chlorhexidine. Microbiological samples were analyzed for 180 days post-therapy.	Metronidazole and amoxicillin were administered for 14 days.	Microbiological samples collected and assessed up to 180 days post-therapy	Antibiotics and chlorhexidine reduced red complex species in subgingival biofilm and saliva. Lower proportions of these species were observed in all oral sites.
Tatjana Ramich et al. (2015) [120]	Observational study	To compare subgingival plaque sampling strategies after combined mechanical and antibiotic periodontal therapy.	Thirty patients with aggressive or severe chronic periodontitis underwent therapy. Plaque was sampled before and after therapy. Analysis was performed for specific bacteria.	SRP was performed in 1 or 2 sessions, followed by antibiotic administration.	Two recovery evaluations conducted: one at 5 days following therapy and the other at 13 to 15 days later.	Deeper sampling post-therapy detected A.a. in 7% of patients vs. none before. *Tannerella forsythia* was detected more in deeper samples.
Marie Dubar et al. (2019) [121]	Observational Study	To investigate protozoans in subgingival biofilm in periodontitis patients and the effects of SRP treatment.	Subgingival microbiota from 30 periodontitis patients pre- and post-SRP, with healthy and pathological site samples, were analyzed. Protozoans and bacteria were identified using PCR and qPCR.	From T0 (recording periodontal parameters from crevicular fluid samples) to 2–5 weeks after (SRP for periodontal patients, and oral hygiene instructions for control group patients)	Reevaluation at T0 + 13–15 weeks.	*Trichomonas tenax* and two subtypes of *Entamoeba gingivalis* were detected in periodontitis patients. ST1 was associated with clinical parameters and bacterial count. After SRP, only *T. tenax* detection significantly decreased. No clear association between protozoan elimination and improvement in pathological sites.
Ibrahimu Mdala (2013) [122]	Observational study	To analyze bacterial counts in chronic periodontitis after various treatments over 2 years.	Subgingival plaque was collected from 176 subjects at different time points post-treatment and analyzed for 40 bacteria using checkerboard hybridization.	SRP over four weekly visits, treating one quadrant at a time. Amoxicillin (AMOX) 500 mg twice daily and metronidazole (MET) 250 mg three times daily for 14 days were started immediately after the first SRP session. Chlorhexidine 0.1% was given during the SRP phase and for 2 weeks following surgery.	Patients monitored at 3, 6, 12, 18, and 24 months post-treatment.	Short-term reductions in red complex bacteria were observed with AMOX + MET + TET treatment. No long-term significant effects were observed with any treatment. Poor oral hygiene diminished treatment effects. Smoking and bleeding on probing were predictors of more red complex counts.

## Data Availability

Not applicable.

## Abbreviations

A.a.	Aggregatibacter actinomycetemcomitans
AgP	aggressive periodontitis
AIAN	American Indian/Alaska Native
AMX	amoxicillin
APT	active periodontal therapy
ARGs	antibiotic-resistance genes
AT	antibiotics and mechanical treatment
BOP	bleeding on probing
CAL	clinical attachment level
C/MIP	Grade C Molar–Incisor Pattern Periodontitis
EMD	enamel matrix derivative
FDIS	full-mouth disinfection
GEE	generalized estimating equation
GI	gingival inflammation
GTR	guided tissue regeneration
HBO	hyperbaric oxygen
HS	healthy state
LPO	lactoperoxidase
MGEs	mobile genetic elements
MRGs	metal-resistance genes
MTZ	metronidazole
NB	negative binomial
NSPT	non-supportive periodontal therapy
OHI	hygiene education
PAD	photoactivated disinfection
PD	probing depth
PRISMA	Preferred Reporting Items for Systematic Reviews and Meta-Analyses
PROSPERO	The International Prospective Register of Systematic Reviews
PS	periodontitis state
RS	resolved state
SDD	sub-antibacterial dose doxycycline
SI	subgingival instrumentation
SRP	scaling and root planing
SRP + MM	scaling and root planing combined with minocycline hydrochloride microspheres
SURG	periodontal surgery
TET	locally administered tetracycline
